# Missing covariates in competing risks analysis

**DOI:** 10.1093/biostatistics/kxw019

**Published:** 2016-05-13

**Authors:** Jonathan W. Bartlett, Jeremy M. G. Taylor

**Affiliations:** Statistical Innovation Group, AstraZeneca Cambridge, UK; Department of Biostatistics, School of Public Health, University of Michigan, Ann Arbor, MI, USA

**Keywords:** Competing risks, Missing covariates, Missing at random, Multiple imputation

## Abstract

Studies often follow individuals until they fail from one of a number of competing failure types. One approach to analyzing such competing risks data involves modeling the cause-specific hazards as functions of baseline covariates. A common issue that arises in this context is missing values in covariates. In this setting, we first establish conditions under which complete case analysis (CCA) is valid. We then consider application of multiple imputation to handle missing covariate values, and extend the recently proposed substantive model compatible version of fully conditional specification (SMC-FCS) imputation to the competing risks setting. Through simulations and an illustrative data analysis, we compare CCA, SMC-FCS, and a recent proposal for imputing missing covariates in the competing risks setting.

## 1. Introduction

In competing risks analysis, individuals are followed up until they “fail” from one of a set of possible causes of failure, e.g. cause-specific death. In such situations, it is often of interest to model how the hazard of failure from the different causes depends on a set of covariates recorded at cohort entry. Arguably, the most direct approach to analyzing competing risks data is to specify models for the cause-specific hazard functions (Andersen *and others*, 2002).

A problem that arises in practice is that one or more covariates contain missing values. While extensive research has been conducted into missing covariates in the context of generalized linear models (Ibrahim *and others*, 2005) and the Cox model for single failure type data (Herring and Ibrahim, 2001; White and Royston, 2009), little has been done on competing risks. Recently, Escarela *and others* (2013) proposed a likelihood-based approach for handling incomplete covariates in competing risks analysis, based on models for the conditional survival distributions. They focused on the case of two partially observed discrete covariates, and developed a copula-based approach to model specification, under both missing at random (MAR) and missing not at random (MNAR) mechanisms (Rubin, 1976).

The simplest and most commonly used approach to handling missing covariates is to fit models of interest excluding those with missing covariate values, in a so-called complete case analysis (CCA). In Section 3, we establish a condition under which CCA is valid, and discuss how the observed data can be used to assess compatibility with this condition. An increasingly popular approach for handling missing data is to use multiple imputation (MI), usually under the MAR assumption (Carpenter and Kenward, 2013). In Section 4, we describe recent proposals for imputing covariates in the competing risks setting using standard software. We then propose an approach that ensures covariates are imputed using models that are compatible with the analyst's specified cause-specific hazard models. We compare CCA with the MI approaches in simulations in Section 5. In Section 6, we apply CCA and MI to handle missing covariates in an analysis of data from the NHANES III study. We conclude with a discussion in Section 7.

## 2. Setup and full data analysis

We assume a sample of $n$ independent individuals. For each, we observe vectors of time-independent baseline covariates $X$ and $Z$. For the moment, we assume both are fully observed. For each individual, we assume the existence of a time to failure $T$ and failure indicator $D^{\ast } \in \{1,\ldots ,K\}$, where $D^{\ast }$ indicates the type of failure. As described by Prentice *and others* (1978), the basic estimable quantities in the competing risks setting are the cause-specific hazard functions. For cause $k$, the cause-specific hazard function is defined as

\[ h_{k}(t\,|\,X,Z) = \lim_{\Delta t \rightarrow 0} P(t \leq T < t+ \Delta t, D^{\ast}=k \,|\, T \geq t, X,Z)/ \Delta t \]

Often the time to failure is censored, and so we further assume the existence of a time to censoring $C$ for each individual. We observe $Y=\min (T,C)$ and $D=1(T<C)D^{\ast }$, which indicates either the observed cause of failure or that the individual is censored ($D=0$). We assume that censoring is independent, in the sense that $(T,D^{\ast }) \perp \!\!\!\perp C\, |\, (X,Z)$. An individual's contribution to the likelihood function, conditional on $X$ and $Z$, is then equal to

(2.1) \begin{align} f(Y,D\,|\,X,Z) & \propto \exp\left[- \int^{Y}_{0} h_{0}(u\,|\,X,Z) \, {\rm d} u \right] [ h_{0}(Y\,|\,X,Z)]^{I(D=0)} \nonumber \\ & \quad \times \prod^{K}_{k=1} \exp\left[- \int^{Y}_{0} h_{k}(u\,|\,X,Z) \, {\rm d} u \right] [ h_{k}(Y\,|\,X,Z) ]^{I(D=k)} \end{align}

where $h_{0}(t\,|\,X,Z)$ denotes the hazard for the censoring process, given $X$ and $Z$. When covariates are fully observed, as described by Prentice *and others* (1978), inference for a particular (say $k$th) cause-specific hazard function can proceed by using standard survival analysis procedures, treating both censoring events and failures from causes other than $k$ as censored at their time of failure. A popular approach is to assume a Cox proportional hazards model

(2.2) \begin{equation*} h_{k}(t\,|\,X,Z) = h_{0k}(t) \exp(g_{k}(X,Z,\beta_{k}))\end{equation*}

where $h_{k}(t|X,Z)$ denotes the cause-specific hazard function for cause $k$, $h_{0k}(t)$ denotes the baseline hazard function for cause $k$, $\beta _{k}$ denotes a vector of cause-specific regression coefficients, and $g_{k}(\cdot )$ denotes a known function, indexed by $\beta _{k}$. The baseline hazard functions $h_{0k}(t)$ can either be assumed to follow a parametric form or as is more commonly done in the absence of missing covariates, left arbitrary. In this case, as in Cox's proportional hazards model, the cumulative baseline hazard $H_{0k}(t) = \int ^{t}_{0} h_{0k}(u) \, {\rm d} u$ can be viewed as an infinite dimensional parameter.

An alternative formulation of the competing risks problem involves postulating the existence of latent failure times for each cause of failure. This formulation and analyses based on it relies on strong untestable assumptions surrounding independence of competing risks (Prentice *and others*, 1978; Andersen *and others*, 2002), and so we do not pursue it further here.

## 3. Complete case analysis

We now consider inference when $X$ is partially observed ($Z$ remains fully observed). We let $R$ denote whether all components of $X$ are observed ($R=1$) or some are missing ($R=0$). Without loss of generality, we assume interest lies in fitting a model for the first cause-specific hazard function. In CCA, we fit a model for this using only those individuals with $X$ completely observed and who therefore have $R=1$. In Appendix A of the Supplementary Materials (available at *Biostatistics* online), we show that this will be valid if ${R \perp \!\!\!\perp (T,D^{\ast }) \,|\, (C,X,Z)}$. This assumption encompasses both MAR mechanisms (e.g. missingness dependent only on $Z$) and MNAR mechanisms (e.g. missingness dependent on $X$, or missingness dependent on $C$).

In the special case of single failure type data (i.e. $K=1$), Rathouz (2007) established sufficient conditions under which CCA gives valid inferences. Specifically, he showed that valid inferences are obtained if $R \perp \!\!\!\perp (T,X) \,|\, (C,Z)$. We note that since single failure time data are a special case of competing risks with $K=1$, our result extends that of Rathouz (2007) in that missingness in $X$ can be dependent on $X$. This extension intuitively makes sense in light of the fact that CCA makes no distinction between which covariates are fully observed and which are partially observed in the full sample.

A special case of the sufficient missingness assumption is when $R \perp \!\!\!\perp (T,D^{\ast },C) \,|\, (X,Z)$, in which case missingness in $X$ is covariate dependent. As discussed by Bartlett *and others* (2014), such an assumption may sometimes be plausible when, as here, the covariates temporally preceed the outcome. This is because in order for $R \,\not \!\perp \!\!\!\perp (T,D^{\ast },C) \,|\, (X,Z)$, there would have to exist another baseline variable $V$ which itself has an independent effect on $(T,D^{\ast },C)$ and on $R$.

As with the MAR assumption, in general, it is not possible to verify the assumption $R \perp \!\!\!\perp (T,D^{\ast }) \,|\, (C,X,Z)$ from the observed data. It is, however, possible to check whether the observed data are compatible with a stronger version of the assumption. Specifically, consider the stronger assumptions that $R \perp \!\!\!\perp (T,D^{\ast },X) \,|\, (C,Z)$ and that $X \perp \!\!\!\perp C \,|\, Z$ (this condition being unnecessary if there is no censoring). Then by ignoring the actual cause of failure, the results of Rathouz (2007) imply that: (1) $C \perp \!\!\!\perp T \,|\, (R=1,X,Z)$, (2) $C \perp \!\!\!\perp X\, |\, (R=1,Z)$, (3) $C \perp \!\!\!\perp T \,|\, (R,Z)$, and (4) $T \perp \!\!\!\perp R\, |\, Z$. One can then check whether the observed data are compatible with these implications of the stronger assumptions. Specifically, (1) implies one can check whether (2) holds by fitting a model for the hazard of censoring (treating failures as censoring events) conditional on $X$ and $Z$ within the complete cases. If the stronger assumptions hold, one should find that the hazard for censoring in this model does not depend on $X$ (i.e. (2) is satisfied). Next, (3) implies that censoring is independent conditional on $(R,Z)$. Thus, (4) can be checked by fitting a model for the hazard of any failure (i.e. combining the failure types), conditional on $R$ and $Z$. If (4) is satisfied, one should find that the hazard of any failure does not depend on $R$, conditional on $Z$. It is important to note, however, that if the observed data are not consistent with the implications of the stronger assumptions, this does not necessarily mean that the CCA is invalid.

## 4. MI assuming MAR

As described in the introduction, MI assuming data are MAR is a commonly adopted approach for handling missing covariates. In this section, we first consider the plausibility of MAR. We then describe a recently proposed MI approach for the competing risks setting. Lastly, we propose an approach that imputes covariates from models which are compatible with the analyst's specified models for the cause-specific hazard functions.

### 4.1. Plausibility of MAR

For the moment, suppose that $X$ is either scalar or a vector of covariates which is either entirely missing or entirely observed. The MAR assumption here means that $R \perp \!\!\!\perp X \,|\, (Y,D,Z)$. MAR is plausible if missingness in $X$ is thought to be dependent on $Z$. Alternatively, if missingness depends on $T$ and/or $D^{\ast }$, then MAR holds in the absence of censoring (since then $Y=T$ and $D=D^{\ast }$). However, if censoring is present, and missingness depends on $T$ and/or $D^{\ast }$, following the results of Rathouz (2007) for time-to-event data, MAR does not hold. Nevertheless, MAR is a useful assumption, since it enables information to be extracted from the incomplete cases, and provides a starting point for possible MNAR sensitivity analyses.

### 4.2. Directly specified imputation models

Imputation models are in practice almost always specified directly as conditional models for the incomplete variable(s), conditional on the fully observed variables. In the present context, this means directly specifying a model for $f(X\,|\,Y,D,Z)$. In the simpler context of incomplete covariates in survival analysis, White and Royston (2009) previously derived imputation models for incomplete covariates which are approximately compatible with a Cox proportional hazards model for the hazard of failure, assuming the latter contains main effects of $X$ and $Z$. Specifically, they proposed that the incomplete $X$ be imputed using an imputation model with $Z$, $D$ (the binary event indicator) and the baseline cumulative hazard function, as covariates. A better approximation additionally includes interactions between $Z$ and the baseline cumulative hazard function. Since the baseline cumulative hazard function is not available prior to analysis, they proposed its approximation by the Nelson–Aalen estimator of the marginal cumulative hazard function. Through simulations, they demonstrated that their approach gives estimates that typically have little or small bias, although larger biases can occur with strong covariate effects.

Recently, Resche-Rigon *and others* (2012) proposed an extension of the results of White and Royston (2009) to the competing risks setting. Assuming Cox proportional hazards models for each cause-specific hazard, they showed using a Taylor series expansion that an approximately compatible imputation model for $X$ uses $Z$, $D$ (as a factor variable) and $H_{0k}(Y)$, $k=1,\ldots ,K$ as covariates. Resche-Rigon *and others* (2012) further showed that this approximation could be improved by including the interactions $Z\times H_{0k}(\cdot )$, $k=1,\ldots ,K$. Since the cumulative baseline hazard functions are not available prior to imputation, they proposed their approximation by the corresponding Nelson–Aalen estimates of the (marginal) cumulative cause-specific hazard functions. Simulation results suggested that the approach led to estimates with little bias, and confidence intervals with nominal coverage. They also demonstrated that applying the approach of White and Royston (2009) treating failures from competing risks which were not of primary interest as censoring, led to bias. When $X$ is vector valued, and there are multiple missingness patterns, Resche-Rigon *and others* (2012) proposed using the fully conditional specification MI approach (van Buuren, 2007).

The approach proposed by Resche-Rigon *and others* (2012) is attractive since it can be readily implemented using existing software for MI. A potential drawback, however, is that the imputation model used is only approximately compatible with the assumed models for the cause-specific hazard functions. It is, therefore, expected that in certain situations (e.g. large covariate effects), the approach may lead to estimates with appreciable biases. Moreover, as described in detail by Bartlett *and others* (2015), more generally it is difficult to choose directly specified imputation models for incomplete covariates that are compatible with outcome models when the incomplete covariates are assumed to have non-linear effects or interactions in the substantive model. These difficulties can, however, be overcome by constructing an imputation model that is compatible with the assumed models for the cause-specific hazard functions.

### 4.3. Substantive model compatible covariate imputation

Suppose for the moment that $X$ is scalar, and is MAR. We further assume that for each cause-specific hazard function, a proportional hazards model conditional on $X$ and $Z$ has been specified, as given in equation (2.2). To ensure the imputation model for $X$ is compatible with the substantive model, we note that $f(X\,|\,Y,D,Z) \propto f(Y,D\,|\,X,Z) f(X\,|\,Z)$. The first part of this is the likelihood contribution given by equation (2.1). Thus a substantive model compatible imputation distribution for $X$ is, up to a constant of proportionality, equal to

(4.1) \begin{equation*} f(X\,|\,Z) \prod^{K}_{k=1} \exp[- \exp\{g_{k}(X,Z,\beta_{k})\} H_{0k}(Y) ] [h_{0k}(Y) \exp\{g_{k}(X,Z,\beta_{k})\} ]^{I(D=k)} \end{equation*}

where we omit the terms corresponding to the censoring process on the assumption that $h_{0}(t\,|\,X,Z)=h_{0}(t\,|\,Z)$. If in a particular application such an assumption is deemed inappropriate, for example based on a preliminary model fit for the censoring process, this can be handled by treating censoring as an additional cause of failure and specifying a proportional hazards model for the censoring process conditional on $X$ and $Z$.

Thus, having specified models for the cause-specific hazards, the imputation distribution specification is completed by specifying a model $f(X\,|\,Z,\phi )$. The model for $f(X\,|\,Z)$ can be chosen to be an appropriate model depending on the variable type of $X$. For example, we may use linear, logistic, ordinal, or multinomial logistic regression models for continuous, binary ordered categorical, and unordered categorical variables, respectively. Count variables can be imputed using Poisson or negative binomial models. In Appendix B.1 of the Supplementary Materials (available at *Biostatistics* online), we describe how a Gibbs sampler can be constructed using this imputation approach, and give details about prior choice. In Appendix B.2 (see supplementary material available at *Biostatistics* online), we describe methods for sampling from the required conditional distributions.

In practice, $X$ is commonly vector valued, with multiple missingness patterns. In this case, a joint model could in principle be specified for $X=(X_{1},\ldots ,X_{p})$, and imputations be drawn from the posterior distribution of the missing data using a Gibbs sampler. One approach in this case is to factorize the joint distribution as a series of univariate conditional models, as proposed by Ibrahim *and others* (1999).

Here, following the popular chained equations or fully conditional specification approach to MI, we instead adopt the substantive model compatible fully conditional specification (SMC-FCS) approach recently proposed by Bartlett *and others* (2015). Rather than specifying a joint model for $f(X\,|\,Z)$, this approach involves specifying, for each partially observed variable $X_{j}$, a model $f(X_{j}\,|\,X_{-j},Z,\phi _{j})$, where $X_{-j}$ denotes the components of $X$ except the $j$th. The partially observed $X_{j}$ are then imputed one at a time. Further details for the algorithm are given in Appendix B.3 of the Supplementary Materials (available at *Biostatistics* online).

The SMC-FCS approach ensures that each partially observed variable is imputed from a model that is compatible with the substantive model, and at the same time permits flexibility since different model types can be specified for each $f(X_{j}\,|\,X_{-j},Z,\phi _{j})$, $j=1,\ldots ,p$. A drawback of the SMC-FCS algorithm is that these models may themselves be mutually incompatible, such that the resulting sampler does not draw imputations from a well-defined Bayesian joint model. However, given recent theoretical developments regarding the properties of standard FCS MI (Liu *and others*, 2013; Hughes *and others*, 2014), we believe the possibility of such incompatibility may not be such a great practical concern for SMC-FCS, provided the models $f(X_{j}\,|\,X_{-j},Z,\phi _{j})$, $j=1,\ldots ,p$ fit well.

## 5. Simulations

In this section, we report the results of simulations to evaluate the performance of CCA and the MI approaches described previously.

### 5.1. Simulation 1: covariate-dependent missingness

For datasets of size $n=1000$, we first generated three covariates $\textbf {X}=(X_{1},X_{2},X_{3})$ as $X_{1} \sim \mbox {Ber}(0.5)$, $X_{2}|X_{1} \sim \mbox {Ber}(0.25+0.50X_{1})$, $X_{3}\,|\,X_{2},X_{1} \sim N(-1+X_{1}+X_{2},1)$. Event times for two competing causes were then generated. The first was generated with hazard $h_{1}(t\,|\,\textbf {X})=0.2 \times 4t^{3} \exp (\textbf {X} \beta _{1})$, with $\beta ^{T}_{1}=(\beta _{11},\beta _{12},\beta _{13})=(1,1,1)$. The second was generated with hazard $h_{2}(t\,|\,\textbf {X})=\exp (\textbf {X} \beta _{2})$, with $\beta ^{T}_{2}=(\beta _{21},\beta _{22},\beta _{23})=(0.5,-1,0.75)$. Censoring times were generated from a uniform distribution between 0.5 and 2. This led to 25% of individuals being censored, 25% failing from cause 1 and 50% from cause 2.

Values in $X_{3}$ were then made missing (at random) with probability $0.25+0.5X_{1}$, leading to 50% missing values. We imputed the missing values in $X_{3}$ using three different directly specified conditional imputation models for $f(X_{3}\,|\,X_{1},X_{2},T,D)$ using the R package MICE. First, following the results of Resche-Rigon *and others* (2012), $X_{3}$ was imputed using a normal linear regression imputation model, using the event indicator $D$ as a categorical predictor, the Nelson–Aalen estimates of the (marginal) cumulative hazard functions (i.e. ignoring covariates), $\hat {H}_{NA1}(Y)$ and $\hat {H}_{NA2}(Y)$, and $X_{1},X_{2}$ as covariates (FCS competing). Secondly, we used an imputation model based on the more accurate approximation derived by Resche-Rigon *and others* (2012), by additionally including interaction terms between each of $X_{1},X_{2}$ and each of $\hat {H}_{NA1}(Y)$ and $\hat {H}_{NA2}(Y)$ (FCS competing int.). Thirdly, to explore the impact of ignoring the second cause of failure at the imputation stage, we also imputed $X_{3}$ as if it were (single failure type) survival data, by treating failures from the second cause as if they were censorings when defining $D$ and calculating $\hat {H}_{NA1}(Y)$, and omitting $\hat {H}_{NA2}(Y)$ from the imputation model (FCS survival). Note that here we did not include the interactions between $X_{1},X_{2}$, and $\hat {H}_{NA1}(Y)$.

Next we imputed $X_{3}$ using the substantive model compatible approach described in Section 4.3, assuming (correctly here) that $X_{3}\,|\,X_{1},X_{2}$ is normal linear regression, and assuming Cox models with linear covariate effects for both causes of failure (SMC-FCS competing). We then imputed again using the substantive model compatible approach, acting as if the data were single failure type data, considering failures only due to cause one (SMC-FCS survival).

For all the imputation methods, five imputations were generated for each dataset. With each imputed dataset, we fitted Cox proportional hazards models for each cause of failure, and combined estimates of the two sets of regression coefficients $\beta _{1}$ and $\beta _{2}$ using Rubin's rules. Using each imputation, we also estimated the cumulative cause-specific hazard function for cause one at $t=0.5$, and obtained standard errors using the R function survfit. These were similarly combined across the five imputations using Rubin's rules.

Table 1 shows the results of the simulations. First, we note the considerable efficiency loss due to missing data as shown by the larger empirical SDs for complete case estimates compared with full data. In line with the results of Section 3, CCA is unbiased since missingness is covariate dependent. Estimates based on FCS MI, accounting for competing risks (FCS competing), showed moderately large biases for most parameters, and consequently low confidence interval coverage for some parameters. This can be attributed to the fact that the imputation model used is only approximately compatible with the cause-specific hazard models, and the baseline cumulative hazards are estimated by the marginal Nelson–Aalen cumulative hazard estimator. The estimate of the first cumulative baseline hazard function at $t=0.5$ was also biased upward. Including interactions between the estimated cumulative hazard functions and $X_{1},X_{2}$ (FCS competing inter) reduced the biases considerably. Moreover, confidence interval coverage was improved, although for $\beta _{13}$ coverage was still poor. In line with the simulation results of Resche-Rigon *and others* (2012), performance was worse when the second cause of failure was treated as if it were censoring (FCS survival), with larger biases and lower confidence interval coverage.

**Table 1.** Mean (empirical SD) of estimates across 1000 simulations, with covariate-dependent missingness in $X_{3}$

Method	$\beta _{11}=1$	$\beta _{12}=1$	$\beta _{13}=1$	$\beta _{21}=0.5$	$\beta _{22}=-1$	$\beta _{23}=0.75$	$\gamma =1.25^\dagger $
	Mean						
Full data	1.01	1.01	1.01	0.50	$-$1.00	0.75	1.25
Complete case	1.02	1.01	1.01	0.50	$-$1.01	0.76	1.24
FCS competing	0.87	1.18	0.59	0.51	$-$0.88	0.62	1.65
FCS compet inter	0.99	1.10	0.75	0.54	$-$0.98	0.68	1.43
FCS survival	0.84	1.21	0.56	0.63	$-$0.70	0.43	1.58
SMC-FCS competing	1.05	1.01	1.00	0.53	$-$1.01	0.75	1.25
SMC-FCS survival	0.83	1.13	1.00	0.75	$-$0.58	0.34	1.13
	SD						
Full data	0.16	0.18	0.08	0.11	0.11	0.05	0.29
Complete case	0.24	0.26	0.13	0.18	0.18	0.07	0.44
FCS competing	0.17	0.19	0.09	0.13	0.13	0.07	0.36
FCS compet inter	0.19	0.20	0.09	0.13	0.13	0.07	0.33
FCS survival	0.17	0.19	0.08	0.12	0.12	0.06	0.35
SMC-FCS competing	0.19	0.21	0.13	0.13	0.14	0.07	0.32
SMC-FCS survival	0.19	0.21	0.13	0.11	0.10	0.04	0.30
	Coverage						
Full data	95	95	96	95	96	94	94
Complete case	95	95	96	97	95	94	92
FCS competing	91	89	11	95	88	66	92
FCS compet inter	97	95	55	94	96	90	98
FCS survival	88	86	3	84	40	2	95
SMC-FCS competing	94	96	95	94	94	95	94
SMC-FCS survival	85	92	94	49	6	0	87

CI indicates empirical coverage of nominal 95% confidence intervals.

$^\dagger \gamma = 100 \times H_{01}(0.5) =1.25$.

Estimates from SMC-FCS accounting for the competing risks showed little bias and confidence interval coverage close or slightly below the nominal 95% level. Of particular note, the cumulative baseline hazard function at $t=0.5$ for the first cause of failure was estimated with little bias, and confidence intervals had only slight under coverage. Comparing empirical standard deviations, we see that SMC-FCS recovers considerable information for the coefficients of the fully observed covariates $X_{1}$ and $X_{2}$, while for the coefficient of the partially observed $X_{3}$ there is no efficiency gain. As expected, imputing treating the second cause of failure as censoring (SMC-FCS survival) led to biased estimates and confidence interval coverage below the nominal level, particularly (as one might expect) for $\beta _{2}$.

**Table 2.** Mean $(SD)$ of estimates across 1000 simulations, with missingness in $X_{2}$ and $X_{3},$ and $X_{2}X_{3}$ interactions present in cause-specific hazard models

Method	$\beta _{11}=1$	$\beta _{12}=1$	$\beta _{13}=1$	$\beta _{14}=-1$	$\beta _{21}=0.5$	$\beta _{22}=-1$	$\beta _{23}=0.75$	$\beta _{24}=1$	$\gamma =1.25^\dagger $
	Mean								
Full data	1.01	1.01	1.01	$-$1.01	0.50	$-$1.01	0.75	1.00	1.23
Complete case	1.07	1.06	1.04	$-$1.05	0.51	$-$1.04	0.77	1.01	1.15
FCS competing	0.79	1.14	0.69	$-$0.45	0.45	$-$0.39	0.73	0.15	1.23
FCS compet inter	0.96	1.12	0.64	$-$0.40	0.53	$-$0.63	0.78	0.31	1.14
FCS survival	0.76	1.27	0.62	$-$0.54	0.48	$-$0.23	0.65	$-$0.01	1.14
SMC-FCS competing	1.02	1.04	1.01	$-$1.02	0.51	$-$1.03	0.77	1.01	1.20
SMC-FCS survival	0.81	1.23	1.01	$-$0.94	0.70	0.02	0.40	$-$0.08	1.12
	SD								
Full data	0.15	0.16	0.10	0.15	0.10	0.12	0.06	0.10	0.34
Complete case	0.37	0.44	0.27	0.39	0.25	0.31	0.16	0.25	0.81
FCS competing	0.17	0.23	0.10	0.11	0.12	0.19	0.08	0.07	0.37
FCS compet inter	0.18	0.27	0.15	0.17	0.13	0.20	0.10	0.09	0.36
FCS survival	0.17	0.23	0.09	0.10	0.11	0.17	0.07	0.07	0.34
SMC-FCS competing	0.19	0.28	0.14	0.27	0.14	0.22	0.10	0.17	0.38
SMC-FCS survival	0.22	0.27	0.14	0.22	0.09	0.08	0.05	0.06	0.36
	Coverage								
Full data	94	96	96	95	96	96	94	94	93
Complete case	94	95	94	95	94	95	94	95	82
FCS competing	83	95	38	26	94	25	97	0	95
FCS compet inter	97	95	51	44	96	61	96	2	89
FCS survival	80	86	14	28	96	5	80	0	89
SMC-FCS competing	94	94	94	96	95	95	94	94	92
SMC-FCS survival	84	87	94	92	60	0	0	0	87

CI indicates empirical coverage of nominal 95% confidence intervals.

$^\dagger \gamma = 100 \times H_{01}(0.5) =1.25$.

### 5.2. Simulation 2: multiple missingness patterns and interactions

In a second set of simulations, we explored imputation of two covariates with multiple missingness patterns, and the ability of the two imputation approaches to accommodate interactions in the competing hazards models. Here $X_{2}$ was made missing with probability $0.75-0.5X_{1}$, while $X_{3}$ was made missing with probability $0.25+0.5X_{1}$, leading to 50% missingness in each variable. The two cause-specific hazard functions were also modified, additionally including the term $X_{2}X_{3}$ in each, with coefficient vectors $\beta _{1}=(1,1,1,-1)$, $\beta _{2}=(0.5,-1,0.75,1)$. This led to 33% of individuals failing due to cause 1, and 67% failing from cause 2. No censoring was imposed.

In the FCS approaches, $X_{2}$ was imputed using logistic regression, conditioning on $X_{1},X_{3}$ and the event indicator and Nelson–Aalen cumulative hazard estimators as before. In “FCS competing inter” as before we included interactions between $X_{1}$ and the Nelson–Aalen cumulative hazard estimates, and similarly between $X_{2}$ ($X_{3}$) and the cumulative hazard estimates when imputing $X_{3}$ (respectively, $X_{2}$). Note, however, that no further modifications were made to attempt to allow for the $X_{2}X_{3}$ interactions in the cause-specific hazard models, with these interaction values simply being passively imputed at the end in the final imputed datasets.

In the SMC-FCS approaches, $X_{2}$ was imputed using a logistic model conditional on $X_{1}$ and $X_{3}$, and the $X_{2}X_{3}$ interactions were included in the cause-specific Cox models. The number of iterations for SMC-FCS was increased from its default of 10 to 20, since MCMC convergence plots of initial simulations suggested more than 10 were required for convergence due to the presence of the interaction term.

Table 2 shows the results. The FCS approaches led to biased estimates and confidence intervals with very poor coverage for the interaction parameters because FCS (at least as implemented here) does not account for the interactions in the cause-specific hazard models. In contrast, SMC-FCS accounting for both competing causes led to valid inferences, while SMC-FCS treating the second cause as censoring as expected led to very biased estimates of $\beta _{2}$ (as expected), although biases for $\beta _{1}$ were smaller.

Three sets of additional simulations are reported in Appendix C of the Supplementary Materials (available at *Biostatistics* online). In the first set, missingness was dependent on $D$, such that CCA was biased, while SMC-FCS gave valid inferences. In the second set, $X_{3}$ was made missing with missingness dependent on $X_{3}$ (MNAR), such that CCA was unbiased, while the MI approaches were biased. In the final set, missingness in $X_{3}$ was again dependent on $X_{1}$, but with the hazard for the second failure type not dependent on $X_{3}$. Here both SMC-FCS approaches were unbiased, with SMC-FCS survival being slightly more efficient.

## 6. Illustrative analysis

To illustrate the two MI approaches, we consider data from the third US National Health and Nutrition Examination Survey (NHANES III), which was conducted between 1988 and 1994. The overall study involved around 40 000 individuals, and consisted of an in-depth survey of their health and nutrition status, obtained from physical examinations and interview. Mortality status at the end of 2011 is available through linkage to the US National Death Index. Here we consider the subset of individuals aged between 60 and 70 at the time of the original survey, which consists of 2583 individuals. By the end of 2011, 1492 (57.8%) had died. Cause of death was classified using the ICD-10 system. For the illustrative analyses, here we focus on how the hazard for death due to cardiovascular disease (CVD) relates to the risk factors shown in Table 3. Here death due to CVD is of primary interest, and deaths due to other causes are competing causes. We categorize deaths as due to CVD, cancer, and other causes, separating out cancer as it represents a large proportion of deaths and may have quite different associations with the risk factors than other causes. There were 358 CVD deaths, 379 cancer deaths, and 755 deaths due to other causes.

We assumed a Cox proportional hazards model for the hazard of death due to CVD, with main effects of each of the risk factors listed in Table 3, and assuming linear effects (on the log hazard scale) of continuous variables. The first column of Table 4 shows estimated log hazard ratios for each risk factor based on the 1106 (42.8%) complete cases. This shows statistically significant evidence for independent associations of each risk factor with hazard of death due to CVD, except for diabetes, with directions of association as expected based on the prior knowledge of CVD. A global test of the proportional hazards assumption using Schoenfeld residuals revealed no evidence ($p=0.77$) against the assumption.

To investigate whether the CCA is valid, following Section 3, we first argue that the assumption that $X \perp \!\!\!\perp C \,|\, Z$ is satisfied here because censoring is almost exclusively due to the length of available follow-up. Next we fitted a Cox model where events were taken as death from any cause, with fully observed sex, age, diabetes (dropping the three observations with diabetes missing) and an indicator $R$ of whether the other risk factors were all available or not, as covariates. Unfortunately, this showed evidence ($p<0.001$) that being a complete case was associated with increased hazard of death, conditional on sex, age, and diabetes. The data are thus not consistent with an assumption that $R \perp \!\!\!\perp (T,D^{\ast },X) \,|\, (C,Z)$. Nevertheless, the CCA may still be valid, if for example missingness in the partially observed covariates is dependent only on $X$ and $Z$. This is arguably quite plausible for variables such as smoking and alcohol consumption.

Next we applied the FCS and SMC-FCS approaches to multiply impute the missing covariate values, using 50 imputations for each method. As in the simulation study, we applied each either accounting for or ignoring (as censoring) failures from causes of death other than the one of interest (CVD).

**Table 3.** Descriptive statistics for baseline risk factors in NHANES III

Variable	Mean (SD)/no. (%)	Number of missing (%)
Sex, female	1302 (50.4)	0
Age (years)	64.4 (2.9)	0
Current smoker	597 (38.9)	1048 (40.6)
Diabetes	427 (16.6)	3 (0.1)
Alcohol consumer$^\dagger $	992 (55.0)	778 (30.1)
Systolic blood pressure (mmHg)	137.8 (19.4)	297 (11.5)
Total cholesterol (mg/dL)	225.6 (45.2)	355 (13.7)
C-reactive protein $>0.21$ mg/dL	946 (42.7)	368 (14.2)
Fibrinogen (mg/dL)	330.8 (96.0)	387 (15.0)

$^{\dagger }$Reported to have had at least 12 alcoholic drinks in the last 12 months.

**Table 4.** Estimated log hazard ratios $(SE)$ for death due to CVD from NHANES III data

				SMC-FCS	SMC-FCS
	Complete case	FCS competing	FCS survival	competing	survival
Male	0.51 (0.18)	0.69 (0.12)	0.69 (0.12)	0.69 (0.12)	0.70 (0.12)
Age (per 10 years)	0.86 (0.27)	0.90 (0.19)	0.91 (0.19)	0.92 (0.19)	0.90 (0.19)
Current smoker	0.59 (0.15)	0.63 (0.13)	0.60 (0.13)	0.63 (0.13)	0.56 (0.13)
Diabetic	0.26 (0.20)	0.74 (0.13)	0.74 (0.13)	0.75 (0.13)	0.75 (0.13)
Alcohol consumer	0.38 (0.16)	0.37 (0.14)	0.38 (0.14)	0.35 (0.14)	0.35 (0.14)
SBP (per 10 mmHg)	0.96 (0.38)	1.38 (0.28)	1.35 (0.28)	1.36 (0.29)	1.35 (0.28)
Cholesterol (mg/mL)	0.34 (0.16)	0.31 (0.12)	0.31 (0.12)	0.31 (0.12)	0.31 (0.12)
CRP ($>$0.21 mg/dL)	0.45 (0.17)	0.45 (0.12)	0.45 (0.13)	0.45 (0.12)	0.44 (0.12)
Fibrinogen (mg/dL)	0.19 (0.08)	0.13 (0.06)	0.13 (0.06)	0.13 (0.06)	0.13 (0.06)

Table 4 shows the estimated log hazard ratios and corresponding standard errors. Estimates and standard errors were very similar across all four MI methods, suggesting that the approximations being made in the directly specified FCS approach are here quite reasonable. The MI standard errors were uniformly smaller than those from CCA, even for the coefficients of fully observed covariates. However, the MI estimates differed materially from the CCA estimates for some risk factors, such as gender, diabetes, and SBP. Unfortunately, we do not believe it is possible to establish here from the observed data whether the CCA assumption or MAR (or neither) is true. From considerations of the nature of the variables, a covariate-dependent MNAR missingness mechanism, under which CCA is valid, is arguably more plausible than MAR.

## 7. Discussion

We have explored approaches for handling missing covariates in competing risks analysis when one is interested in modeling the cause-specific hazard functions. We have shown under what assumptions CCA is valid, and suggested how the observed data can be checked for compatibility with a stronger version of this assumption. Even when CCA is valid, it is however inefficient. Recently Bartlett *and others* (2014) developed an approach for improving upon the efficiency of CCA for conditional mean models when a covariate-dependent MNAR mechanism is assumed, and further work is warranted to extend this to survival and competing risks settings.

Under an MAR assumption, we have proposed a flexible approach to multiply impute missing covariates in competing risks data, based on proportional hazards models for cause-specific hazards. The approach automatically handles user-specified covariate effects in these models, including interactions and non-linear covariate effects. Through simulation we have demonstrated its good finite sample performance, for both the regression coefficients indexing models for cause-specific hazards and for estimation of the cumulative cause-specific baseline hazard functions. In contrast, we have empirically shown that directly specified approximately compatible imputation models in general lead to biased estimates.

The SMC-FCS approach we have described relies on the analyst specifying appropriate models for the cause-specific hazard functions and the covariate models $f(X_{j}\,|\,X_{-j},Z,\phi _{j})$. The assessment of model fit in the context of MI approaches, or indeed when data are incomplete more generally, is challenging. In the present setting, we would recommend that analysts assess the fit of the covariate $f(X_{j}\,|\,X_{-j},Z,\phi _{j})$ models fitted to those corresponding complete cases. While these fits may themselves be biased (when missingness is not completely at random), if the model appears to fit well in the complete cases, it is arguably plausible that the models are reasonable for the entire sample. For the cause-specific hazard models, if missingness can be assumed to be at most covariate dependent, then again model assessment and selection could be applied to corresponding complete case fits prior to imputation of missing covariates. Alternatively, one could impute missing covariates using SMC-FCS, and then apply model diagnostics for the cause-specific hazard models to the imputed datasets. The obvious limitation with such a strategy is that the missing covariates will have been imputed assuming that the analyst's specified cause-specific models are correctly specified, which would be expected to weaken the potential to detect misspecification in the cause-specific hazard models.

In the context of single failure time data, Qi *and others* (2010) found that using directly specified conditional MI methods for missing covariates gave estimates with large bias when the partially observed covariate was related to the censoring time. Our results explain their finding, and show that if $X$ and $C$ are related, the censoring process must be modeled as an additional competing risk when imputing missing covariates.

Often in competing risks settings, primary interest will be in modeling the hazard of failure due to just one cause. In this case, in the absence of missing covariates, models need not be specified for the causes of failure which are not of interest. An advantage of CCA is that similarly a model need only be specified for the cause(s) of interest. In contrast, if missing covariates are imputed, models must be specified for these causes, (unless the analyst is willing to assume that the cause-specific hazards for the causes not of interest are unrelated to $X$ conditional on $Z$). In this situation, one must choose how to define the competing causes. At one extreme, all of the causes of failure that are not of interest could be combined to form a second cause of failure (in addition to the cause of interest). However, this may be statistically inefficient when the partially observed covariate(s) have different effects on the causes that have been combined. Moreover, if missingness in $X$ is related to failure type, amalgamating the causes not of interest into a single cause may render the MAR assumption invalid, leading to biased estimates.

A closely related approach to handling missing covariates is to fit a single Bayesian joint model, allowing for missingness in the covariates, as described in the case of single failure type data by Chen *and others* (2006). The strengths of such an approach are that one uses a coherent joint model for the data, and uses well-defined priors for all model parameters. However, with multiple partially observed variables, arguably specifying joint models becomes more challenging. Moreover, the Gibbs sampler developed by Chen *and others* (2006) is more involved than the SMC-FCS algorithm, and unlike SMC-FCS, is not currently available in software.

A further alternative approach to handling missing data is based on inverse probability weighting (IPW). IPW and doubly robust estimators assuming MAR have been developed for the Cox model with single failure time data (Wang and Chen, 2001; Qi *and others*, 2010), and further work is warranted on extending these to the competing risks setting. Lastly, we note that an alternative approach to competing risks analysis is based on modeling covariate effects on the cumulative incidence function (Fine and Gray, 1999), and further research is similarly warranted to explore missing covariates within this framework.

## Supplementary materials

Supplementary Material is available at http://biostatistics.oxfordjournals.org.

## Funding

This work was supported by a UK Medical Research Council Fellowship (MR/K02180X/1) [to J.W.B.] and by grant CA129102 from the US National Institutes of Health [to J.M.G.T.]. Funding to pay the Open Access publication charges for this article was provided by the UK Medical Research Council.

## Supplementary Material

Supplementary Data
