# Supplementary material for: Missing covariates in competing risks analysis
Source: Biostatistics. 2016 May 13;17(4):751–63. doi: 10.1093/biostatistics/kxw019 (PMC5031948; doi:10.1093/biostatistics/kxw019)
Supplement: Supplementary Data [file supp_17_4_751__index.html]

Missing covariates in competing risks analysis — Supplementary Data 

# Missing covariates in competing risks analysis

## Supplementary Data

Supplementary Data

**Files in this Supplementary Material:**

- Supplementary Data
