# Supplementary material for: Missing covariates in competing risks analysis
Source: Biostatistics. 2016 May 13;17(4):751–63. doi: 10.1093/biostatistics/kxw019 (PMC5031948; doi:10.1093/biostatistics/kxw019)
Supplement: Supplementary Data [file supp_kxw019_kxw019supp.pdf]

Biostatistics (2016), **0**, 0, *pp.* 1–12
doi:10.1093/biostatistics/supp˙materials˙2016˙04˙01

Missing covariates in competing risks analysis Supplementary Materials

JONATHAN W. BARTLETT*

Statistical Innovation Group, AstraZeneca, Riverside 2, Granta Park, Cambridge, CB21 6GP, UK

jwb133@googlemail.com

JEREMY M.G. TAYLOR

Department of Biostatistics, School of Public Health, University of Michigan, MI, Ann Arbor, U.S.A.

© The Author 2016. Published by Oxford University Press. All rights reserved. For permissions, please e-mail: journals.permissions@oup.com

APPENDIX

A. Validity of complete case analysis

Without loss of generality, we consider the cause specific hazard function for the first cause in the complete cases, $h_1(t|X, Z, R = 1)$. This is equal to

$$
\begin{aligned}
&\lim_{h\to 0} \frac{1}{h} P(t \leqslant Y < t+h, D=1|R=1, X, Z, Y \geqslant t) \\
&= \lim_{h\to 0} \frac{1}{h} \frac{P(t \leqslant T < t+h, D^*=1, C \geqslant t, R=1|X, Z)}{P(R=1, T \geqslant t, C \geqslant t|X, Z)} \\
&= \lim_{h\to 0} \frac{1}{h} \frac{P(t \leqslant T < t+h, D^*=1, C \geqslant t|X, Z)}{P(T \geqslant t, C \geqslant t|X, Z)} \times \frac{P(R=1|t \leqslant T < t+h, D^*=1, C \geqslant t, X, Z)}{P(R=1|T \geqslant t, C \geqslant t, X, Z)} \\
&= \lim_{h\to 0} \frac{1}{h} \frac{P(t \leqslant T < t+h, D^*=1|X, Z)P(C \geqslant t|X, Z)}{P(T \geqslant t|X, Z)P(C \geqslant t|X, Z)} \times \frac{P(R=1|T=t, D^*=1, C \geqslant t, X, Z)}{P(R=1|T \geqslant t, C \geqslant t, X, Z)} \\
&= \lim_{h\to 0} \frac{1}{h} \frac{P(t \leqslant T < t+h, D^*=1|X, Z)}{P(T \geqslant t|X, Z)} \times \frac{P(R=1|T=t, D^*=1, C \geqslant t, X, Z)}{P(R=1|T \geqslant t, C \geqslant t, X, Z)} \\
&= h_1(t|X, Z) \times \frac{P(R=1|T=t, D^*=1, C \geqslant t, X, Z)}{P(R=1|T \geqslant t, C \geqslant t, X, Z)} \qquad \text{(A.1)}
\end{aligned}
$$

A complete case analysis will thus give valid inferences when the second term in the preceding equation is equal to one. An obvious sufficient condition for this to hold is that missingness is covariate dependent, in the sense that $R \perp\!\!\!\perp (T, D^*, C)|(X, Z)$.

We now show that a weaker sufficient condition is that $R \perp\!\!\!\perp (T, D^*)|(C, X, Z)$, which permits missingness to depend on the time to censoring C, in addition to X and Z. To this end, we first show that under this assumption censoring remains independent in the complete cases ($(T, D^*) \perp\!\!\!\perp C|(X, Z, R = 1)$), utilizing the assumption that $(T, D^*) \perp\!\!\!\perp C|(X, Z)$:

$$
\begin{aligned}
P(T, D^*, C|X, Z, R=1) &= \frac{P(T, D^*, C, R=1|X, Z)}{P(R=1|X, Z)} \\
&= \frac{P(R=1|T, D^*, C, X, Z)P(T, D^*, C|X, Z)}{P(R=1|X, Z)} \\
&= \frac{P(R=1|C, X, Z)P(T, D^*|X, Z)P(C|X, Z)}{P(R=1|X, Z)} \\
&= P(T, D^*|X, Z)P(C|X, Z, R=1)
\end{aligned}
$$

Using these results we then have that the second term in equation (A.1) is equal to

$$\begin{aligned}\frac{P(R=1|T=t,D^*=1,C\geqslant t,X,Z)}{P(R=1|T\geqslant t,C\geqslant t,X,Z)} &= \frac{\frac{P(T=t,D^*=1,C\geqslant t|X,Z,R=1)P(R=1|X,Z)}{P(T=t,D^*=1,C\geqslant t|X,Z)}}{\frac{P(T\geqslant t,C\geqslant t|X,Z,R=1)P(R=1|X,Z)}{P(T\geqslant t,C\geqslant t|X,Z)}} \\ &= \frac{\frac{P(T=t,D^*=1|X,Z)P(C\geqslant t|X,Z,R=1)}{P(T=t,D^*=1|X,Z)P(C\geqslant t|X,Z)}}{\frac{P(T\geqslant t|X,Z)P(C\geqslant t|X,Z,R=1)}{P(T\geqslant t|X,Z)P(C\geqslant t|X,Z)}} \\ &= 1\end{aligned}$$

such that the complete case analysis is valid.

B. Multiple imputation

B.1 *Gibbs sampler and prior choice*

Multiple imputation as originally conceived consists of imputing missing data as draws from the posterior distribution of the missing data given the observed. This predictive distribution is based on a Bayesian model - i.e. a model for the full data plus specification of prior distributions for the model parameters. Most often the required posteriors are not available in closed form. In such cases, we can use Gibbs sampling to draw from the required posteriors, which involves repeatedly sampling from each of the fully conditional distributions (of parameters and missing data). In the present context, assuming independent priors for the parameters in each cause specific model, the Gibbs sampler consists of drawing from the following distributions at each iteration:

$$f(\beta_k, H_{0k}(.)|y, d, z, x^{obs}, x^{mis}),\ k = 1, .., K$$

$$f(\phi|z, x^{obs}, x^{mis})$$

$$f(x^{mis}|y, d, z, \phi, \beta_1, H_{01}(.), .., \beta_K, H_{0K}(.))$$

where lower case letters denote the observed values of the corresponding random variables across all individuals, and x^{obs} and x^{mis} denote the observed and current imputations of X across all individuals.

As typically implemented, MI algorithms make use of non-informative priors for all model parameters. In the present context, we adopt improper flat priors for the regression coefficients β_k, $k = 1, .., K$. If the cause specific baseline hazard functions are specified parametrically, then default non-informative priors can be chosen for the corresponding parameters. Often however one wishes to make no assumption about the baseline hazards, such that $H_{0k}(.)$, $k = 1, .., K$ are infinite dimensional parameters.

In the simpler setting of single failure time data, Chen *and others* (2006) have developed theory for the propriety of the posterior distributions for the Cox model, including where some covariates are missing, based on using a gamma process prior for the (assumed arbitrary) cumulative baseline hazard function. Based on this they developed a Gibbs sampling algorithm for sampling from the required posterior distributions. In the same setting, Bartlett *and others* (2015) recently proposed a simpler Gibbs sampling scheme, in which at each iteration, first a new draw of β_1 is sampled from a multivariate normal distribution with mean and covariance as given by Cox's partial likelihood. As shown by Kalbfleisch (1978), whose results were later extended by Sinha *and others* (2003), this approximate posterior for β_1 can be justified by assuming a very diffuse gamma process prior on the cumulative baseline hazard. The algorithm proposed by Bartlett *and others* (2015) then updates the cumulative baseline hazard by calculating the usual Breslow estimator, conditional on the value of β_1 sampled in the preceding step. This approach thus ignores uncertainty in the cumulative baseline hazard function. Nonetheless, in simulations, Bartlett *and others* (2015) obtained satisfactory confidence interval coverage for estimation of β_1.

We extend the approach described by Bartlett *and others* (2015) to the competing risks setting. Specifically, for each $k = 1, .., K$, we first draw β_k from an approximate multivariate normal posterior, with mean and covariance based on the corresponding partial likelihood. We then update $H_{0k}(.)$ using the Breslow estimator, conditioning on the drawn value of β_k. We explore the

finite sample performance of this approach, and in particular also investigate the performance for inferences about $H_{0k}(.)$, in the simulation studies.

B.2 *Sampling methods*

We now describe how missing values in X can be sampled, considering separately the case of categorical covariates and non-categorical covariates.

Categorical covariates If X has a finite sample space, we can directly sample from the imputation distribution. Specifically, let k be the constant of proportionality such that

$$P(X|Z,Y,D) = kf(Y,D|X,Z)P(X|Z)$$

Without loss of generality suppose that X has sample space $\{1,..,S\}$, such that

$$1 = \sum_{s=1}^{S} kf(Y,D|X=s,Z)P(X=s|Z)$$

Then we have

$$k = \frac{1}{\sum_{s=1}^{S} f(Y,D|X=s,Z)P(X=s|Z)}$$

and $P(X=s'|Z,Y,D)$ is equal to

$$\frac{f(Y,D|X=s',Z)P(X=s'|Z)}{\sum_{s=1}^{S} f(Y,D|X=s,Z)P(X=s|Z)}$$
$$= \frac{\prod_{k=1}^{K} \exp\left[-\exp\left\{g_k(X=s',Z,\beta_k)\right\} H_{0k}(Y)\right] \left[\exp\left\{g_k(X=s',Z,\beta_k)\right\}\right]^{I(D=k)} P(X=s'|Z)}{\sum_{s=1}^{S} \prod_{k=1}^{K} \exp\left[-\exp\left\{g_k(X=s,Z,\beta_k)\right\} H_{0k}(Y)\right] \left[\exp\left\{g_k(X=s,Z,\beta_k)\right\}\right]^{I(D=k)} P(X=s|Z)}$$

Other covariate types More generally, rejection sampling can be used to draw from the distribution, using $f(X|Z)$ as the proposal distribution. To use rejection sampling the ratio of the target density to the proposal density must be bounded above, up to a constant of proportionality, by a quantity not involving X. From equation 3.1 of the main paper, here this ratio is simply equal to $f(Y,D|X,Z)$.

First suppose that $D = 0$, such that an individual is censored at time Y. Then we have $f(Y, D = 0|X, Z) \leqslant S_0(Y|Z)h_0(Y|Z)$. To sample a missing value of X^*, we sample from $f(X|Z)$, sample $U \sim U(0, 1)$, and accept X^* if

$$\begin{aligned} U &\leqslant \frac{f(Y, D = 0|X^*, Z)}{S_0(Y|Z)h_0(Y|Z)} \\ &= \prod_{k=1}^{K} \exp\left[-\exp\left\{g_k(X^*, Z, \beta_k)\right\} H_{0k}(Y)\right] \end{aligned}$$

Now suppose that $D > 0$. Then we have

$$\begin{aligned} f(Y, D|X, Z) &\leqslant S_0(Y|Z) \exp\left[-\exp\left\{g_D(X, Z, \beta_D)\right\} H_{0D}(Y)\right] h_{0D}(Y) \exp\left\{g_D(X, Z, \beta_D)\right\} \\ &= S_0(Y|Z)h_{0D}(Y) \exp\left[g_D(X, Z, \beta_D) - \exp\left\{g_D(X, Z, \beta_D)\right\} H_{0D}(Y)\right] \end{aligned}$$

Differentiation with respect to $g_D()$ shows that the expression takes its maximum value when $\exp\left\{g_D(X, Z, \beta_D)\right\} H_{0D}(Y) = 1$, so that

$$f(Y, D|X, Z) \leqslant S_0(Y|Z)h_{0D}(Y) \frac{\exp(-1)}{H_{0D}(Y)}$$

To sample a missing value of X^*, we sample from $f(X|Z)$, sample $U \sim U(0, 1)$, and accept X^* if

$$\begin{aligned} U &\leqslant f(Y, D|X^*, Z) \frac{\exp(1)H_{0D}(Y)}{S_0(Y|Z)h_{0D}(Y)} \\ &= H_{0D}(Y) \exp\{1 + g_D(X^*, Z, \beta_D)\} \prod_{k=1}^{K} \exp\left[-\exp\left\{g_k(X^*, Z, \beta_k)\right\} H_{0k}(Y)\right] \end{aligned}$$

B.3 *Multiple missingness patterns*

Here we describe the SMC-FCS algorithm in the case of multiple partially observed covariates and multiple missingness patterns. For each partially observed variable X_j, we specify a model $f(X_j|X_{-j}, Z, \phi_j)$, where X_{-j} denotes the components of X except the jth. Each iteration of the SMC-FCS algorithm then consists of sampling the missing values in X_j, for $j = 1, .., p$. To impute

the missing values in X_j we draw sequentially from:

$$
\begin{aligned}
& f(\beta_1, H_{01}(.)|t, d, z, x^{obs}, x^{mis}) \\
& \vdots \\
& f(\beta_K, H_{0K}(.)|t, d, z, x^{obs}, x^{mis}) \\
& f(\phi_j|z, x^{obs}, x^{mis}) \\
& f(x_j^{mis}|t, d, z, x_{-j}, \phi, \beta_1, H_{01}(.), .., \beta_K, H_{0K}(.))
\end{aligned}
$$

where x_{-j} denotes the current values of X_{-j} across all individuals, which consists of observed values and current imputed values.

C. Additional simulations

C.1 *Simulation 3 - missingness dependent on failure type*

In a third set of simulations (Table 1), we made values of X_3 missing with probability $0.2 + 0.3D$, where $D = 1, 2$ (failure due to cause 1, failure due to cause 2), leading to approximately 50% missing values. Such a mechanism could be induced if missingness in X_3 was driven by an unobserved baseline variable V, which itself is an independent predictor of failure type. Here CCA is biased, since missingness is dependent on D. For the MI approaches, results were broadly similar to the first simulation set, except that the biases of the FCS approaches were smaller, leading to improved confidence interval coverage. Nonetheless, estimates of β_{13} remained biased using FCS accounting for competing risks (both with and without the additional interaction terms). In contrast, estimates based on SMC-FCS accounting for competing risks again led to unbiased estimates. Interestingly, SMC-FCS treating failures from the second cause as censoring events gave estimates for β_1 with little bias, and confidence interval coverage fairly close to the nominal level.

C.2 *Simulation 4 - covariate dependent MNAR*

Table 2 shows results from simulations where X_3 was made missing with probability $\text{expit}(0.75X_3)$, resulting in a covariate dependent MNAR mechanism. As expected CCA is unbiased, whereas the MI approaches, which assume MAR, are biased. Nevertheless, the biases in 'SMC-FCS competing' are arguably modest, with the exception being the cumulative baseline hazard function estimate at $t = 0.5$.

C.3 *Simulation 5 - X_3 conditionally independent of hazard for second cause of failure*

Table 3 shows results from simulations where X_3 was once again made missing (at random) with probability $0.25 + 0.5X_1$, but with $\beta_2^T = (\beta_{21}, \beta_{22}, \beta_{23}) = (0.5, -1, 0)$, such that the partially observed covariate X_3 had no independent effect on the hazard from the second cause of failure. As expected, the 'FCS competing' and 'FCS survival' approaches now had similar performance, but they remained substantially biased for β_{13}, with zero coverage of confidence intervals for this parameter. Estimates based on 'FCS competing int.' as before had reduced bias, but estimates for β_{13} remained materially biased, and confidence interval coverage was low. SMC-FCS allowing for competing risks was again approximately unbiased with good confidence interval coverage, although the estimate of $H_{01}(0.5)$ was biased upwards somewhat. As expected due to X_3 having no independent effect on the second cause hazard, SMC-FCS treating failures from the second cause as censoring events led to unbiased estimates, and estimates for β_{23} that were more efficient than 'SMC-FCS competing', which here allowed for the possibility that $\beta_{23} \neq 0$. Confidence interval coverage for β_{23} from 'SMC-FCS survival' was above the nominal level, which is consistent with existing theory on the performance of Rubin's variance estimator when the imputer makes an assumption (here that $\beta_{23} = 0$) that the analyst does not (Meng (1994)).

References

Bartlett, J W, Seaman, S R, White, I R and Carpenter, J R. (2015). Multiple imputation of covariates by fully conditional specification: Accommodating the substantive model. *Statistical Methods in Medical Research* **24**, 462–487.

Chen, M, Ibrahim, J G and Shao, Q. (2006). Posterior propriety and computation for the Cox regression model with applications to missing covariates. *Biometrika* **93**, 791–807.

Kalbfleisch, J D. (1978). Non-parametric Bayesian analysis of survival time data. *Journal of the Royal Statistical Society B* **40**, 214–221.

Meng, X L. (1994). Multiple-imputation inferences with uncongenial sources of input (with discussion). *Statistical Science* **10**, 538–573.

Sinha, D, Ibrahim, J G and Chen, M. (2003). A Bayesian justification of Cox's partial likelihood. *Biometrika* **90**, 629–641.

Table 1. Mean (SD) of estimates across 1,000 simulations, with missingness dependent on failure indicator D. CI indicates empirical coverage of nominal 95% confidence intervals

Method	$\beta_{11} = 1$	$\beta_{12} = 1$	$\beta_{13} = 1$	$\beta_{21} = 0.5$	$\beta_{22} = -1$	$\beta_{23} = 0.75$	$\gamma = 1.25$ *
			Mean				
Full data	1.00	1.01	1.01	0.51	-1.00	0.75	1.24
Complete case	0.98	0.84	1.02	0.55	-1.38	0.86	1.85
FCS competing	0.93	0.98	0.84	0.49	-0.97	0.76	1.62
FCS compet inter	0.98	1.05	0.90	0.51	-1.03	0.76	1.35
FCS survival	0.95	1.13	0.66	0.53	-0.83	0.64	1.54
SMC-FCS competing	0.99	1.01	1.00	0.51	-0.99	0.74	1.26
SMC-FCS survival	0.95	1.00	1.05	0.80	-0.56	0.28	1.28
			SD				
Full data	0.13	0.14	0.07	0.11	0.11	0.05	0.26
Complete case	0.14	0.16	0.08	0.23	0.24	0.10	0.45
FCS competing	0.14	0.15	0.08	0.12	0.13	0.08	0.33
FCS compet inter	0.13	0.15	0.08	0.12	0.14	0.08	0.28
FCS survival	0.13	0.14	0.07	0.11	0.12	0.07	0.31
SMC-FCS competing	0.14	0.15	0.09	0.12	0.14	0.08	0.28
SMC-FCS survival	0.14	0.16	0.09	0.10	0.11	0.04	0.29
			Coverage				
Full data	96	95	93	97	95	95	94
Complete case	96	83	94	95	60	82	84
FCS competing	95	96	73	96	95	94	93
FCS compet inter	97	95	85	96	94	94	97
FCS survival	95	88	7	96	77	71	93
SMC-FCS competing	97	94	95	96	94	94	94
SMC-FCS survival	94	96	90	23	3	0	94

*$\gamma = 100 \times H_{01}(0.5) = 1.25$

Table 2. Mean (SD) of estimates across 1,000 simulations, with missingness in X_3 dependent on X_3. CI indicates empirical coverage of nominal 95% confidence intervals

Method	$\beta_{11} = 1$	$\beta_{12} = 1$	$\beta_{13} = 1$	$\beta_{21} = 0.5$	$\beta_{22} = -1$	$\beta_{23} = 0.75$	$\gamma = 1.25$ *
			Mean				
Full data	1.00	1.02	1.00	0.51	-1.00	0.75	1.23
Complete case	1.00	1.02	1.01	0.50	-1.01	0.76	1.22
FCS competing	0.93	1.08	0.70	0.58	-0.83	0.63	1.94
FCS compet inter	0.97	1.10	0.81	0.57	-0.89	0.66	1.70
FCS survival	0.95	1.14	0.61	0.61	-0.75	0.55	1.88
SMC-FCS competing	0.99	1.06	0.95	0.57	-0.89	0.68	1.59
SMC-FCS survival	0.94	1.08	1.00	0.78	-0.57	0.33	1.43
			SD				
Full data	0.13	0.14	0.07	0.11	0.11	0.05	0.25
Complete case	0.18	0.18	0.10	0.16	0.17	0.08	0.39
FCS competing	0.13	0.14	0.08	0.12	0.12	0.08	0.38
FCS compet inter	0.14	0.14	0.08	0.12	0.13	0.08	0.34
FCS survival	0.13	0.14	0.07	0.12	0.11	0.07	0.37
SMC-FCS competing	0.14	0.15	0.10	0.12	0.13	0.08	0.34
SMC-FCS survival	0.15	0.15	0.10	0.10	0.10	0.04	0.31
			Coverage				
Full data	94	94	95	96	95	95	94
Complete case	95	95	94	96	95	95	91
FCS competing	94	93	15	91	74	67	67
FCS compet inter	96	91	51	90	86	79	88
FCS survival	94	88	2	85	50	28	71
SMC-FCS competing	94	93	89	92	88	83	92
SMC-FCS survival	91	91	94	32	3	0	96

*$\gamma = 100 \times H_{01}(0.5) = 1.25$

Table 3. Mean (SD) of estimates across 1,000 simulations, with hazard of second cause (conditionally) independent of X_3. CI indicates empirical coverage of nominal 95% confidence intervals

Method	$\beta_{11} = 1$	$\beta_{12} = 1$	$\beta_{13} = 1$	$\beta_{21} = 0.5$	$\beta_{22} = -1$	$\beta_{23} = 0$	$\gamma = 1.25$ *
			Mean				
Full data	1.00	1.00	1.00	0.50	-1.00	0.00	1.27
Complete case	1.00	1.00	1.01	0.51	-1.01	0.00	1.26
FCS competing	0.88	1.02	0.52	0.57	-0.94	-0.07	2.42
FCS compet inter	1.01	0.98	0.72	0.50	-1.01	0.01	1.78
FCS survival	0.88	1.03	0.52	0.57	-0.94	-0.07	2.42
SMC-FCS competing	1.03	1.00	0.99	0.50	-1.00	0.00	1.32
SMC-FCS survival	1.04	1.00	1.00	0.50	-1.00	0.00	1.29
			SD				
Full data	0.13	0.14	0.06	0.11	0.12	0.05	0.25
Complete case	0.20	0.19	0.10	0.18	0.17	0.06	0.38
FCS competing	0.15	0.14	0.06	0.12	0.12	0.06	0.43
FCS compet inter	0.16	0.15	0.06	0.11	0.13	0.06	0.35
FCS survival	0.15	0.14	0.06	0.12	0.12	0.06	0.43
SMC-FCS competing	0.17	0.17	0.10	0.11	0.12	0.06	0.33
SMC-FCS survival	0.17	0.16	0.09	0.10	0.11	0.04	0.31
			Coverage				
Full data	96	94	95	96	94	93	95
Complete case	96	94	94	94	94	94	93
FCS competing	91	96	0	92	91	80	17
FCS compet inter	97	96	17	95	95	94	87
FCS survival	90	97	0	92	92	82	18
SMC-FCS competing	95	95	96	96	94	94	94
SMC-FCS survival	94	95	95	96	96	99	94

*$\gamma = 100 \times H_{01}(0.5) = 1.25$
